# Abscess Adjacent to the Vas Deferens Mimicking Recurrent Inguinal Hernia: A Case Report and Review of the Literature

**DOI:** 10.7759/cureus.113038

**Published:** 2026-07-20

**Authors:** Ines Ari, Vito De Blasi

**Affiliations:** 1 Surgery, Université Libre de Bruxelles, Brussels, BEL; 2 Surgical Gastroenterology, Centre Hospitalier de Luxembourg, Luxembourg, LUX

**Keywords:** deferentitis, groin mass, inguinal hernia mimic, para-deferential abscess, postoperative complication, spermatic cord abscess, surgical exploration, urogenital infection, vas deferens

## Abstract

Abscesses adjacent to the vas deferens are exceptionally rare and may clinically and radiologically mimic an incarcerated or recurrent inguinal hernia. We report the case of an 82-year-old man with a history of bilateral inguinal hernia repair, recurrent prostatitis, prostate surgery, and atrial fibrillation who presented with a two-day history of painful, non-reducible left inguinoscrotal swelling. Initial ultrasonography demonstrated a non-reducible fat-containing inguinal lesion with preserved Doppler flow, suggestive of an incarcerated inguinal hernia. Non-contrast computed tomography confirmed a left inguinal fat-containing mass associated with ipsilateral ureterohydronephrosis secondary to distal ureteral stenosis.

Because of the strong clinical and radiological suspicion of an incarcerated recurrent inguinal hernia, surgical exploration was undertaken. No recurrent hernia was identified. Instead, a firm necrotic inflammatory mass adjacent to the vas deferens was found and completely excised. Histopathological examination demonstrated acute suppurative inflammation with abscess formation and no evidence of malignancy. Intraoperative cultures grew *Enterococcus faecalis*, whereas anaerobic cultures remained sterile. The postoperative course was uneventful, with complete clinical recovery and no recurrence during follow-up.

This case highlights an important diagnostic pitfall, as inflammatory peri-deferential phlegmon may be misinterpreted as a fat-containing inguinal hernia on imaging. In elderly patients with previous urological disease or recurrent prostatitis presenting with painful groin swelling, uncommon infectious conditions involving the vas deferens or spermatic cord should be considered in the differential diagnosis. Although imaging is essential for the initial assessment, definitive diagnosis often requires surgical exploration together with histopathological and microbiological evaluation.

## Introduction

Acute painful groin swelling is one of the most common surgical emergencies and is most frequently caused by an incarcerated or recurrent inguinal hernia. However, a broad spectrum of conditions arising from structures within the inguinal canal may present with similar clinical features, including inflammatory, infectious, vascular, and neoplastic disorders [1-3]. Because their clinical presentation often overlaps with that of an incarcerated hernia, these uncommon entities may lead to diagnostic uncertainty and unnecessary surgical exploration.

Among these conditions, infections involving the vas deferens and spermatic cord are exceptionally rare. Vasitis (deferentitis) refers to inflammation of the vas deferens itself, whereas funiculitis and spermatic cord abscesses involve the surrounding spermatic cord structures [4-9]. In contrast, the lesion described in the present case was located adjacent to the vas deferens. Histopathological examination confirmed abscess formation without evidence of inflammatory involvement of the vas deferens wall. To our knowledge, this specific anatomical presentation has rarely been reported in the English-language literature.

Published reports of suppurative inflammation involving the vas deferens or spermatic cord are limited to isolated case reports and small case series [4-11]. Most patients present with acute groin pain and an irreducible inguinal mass, leading to an initial diagnosis of incarcerated inguinal hernia. Consequently, the correct diagnosis is frequently established only during surgical exploration and confirmed by histopathological examination.

We report a rare case of an abscess adjacent to the vas deferens that initially mimicked an incarcerated recurrent inguinal hernia. In addition, we review the available literature and discuss the diagnostic challenges, imaging findings, and differential diagnosis of this uncommon entity.

## Case presentation

An 82-year-old man presented to the emergency department with a two-day history of painful, non-reducible swelling of the left inguinoscrotal region. His medical history included bilateral inguinal hernia repair in 2024, recurrent prostatitis, prostate surgery, and atrial fibrillation treated with apixaban. He denied fever, vomiting, or urinary symptoms.

On physical examination, the patient was afebrile and hemodynamically stable. A tender, erythematous, non-reducible left inguinoscrotal swelling was noted without signs of peritonitis. Laboratory investigations demonstrated mild inflammatory syndrome, with a white blood cell count of 13.3 × 10⁹/L and a C-reactive protein level of 28 mg/L.

**Figure 1:**
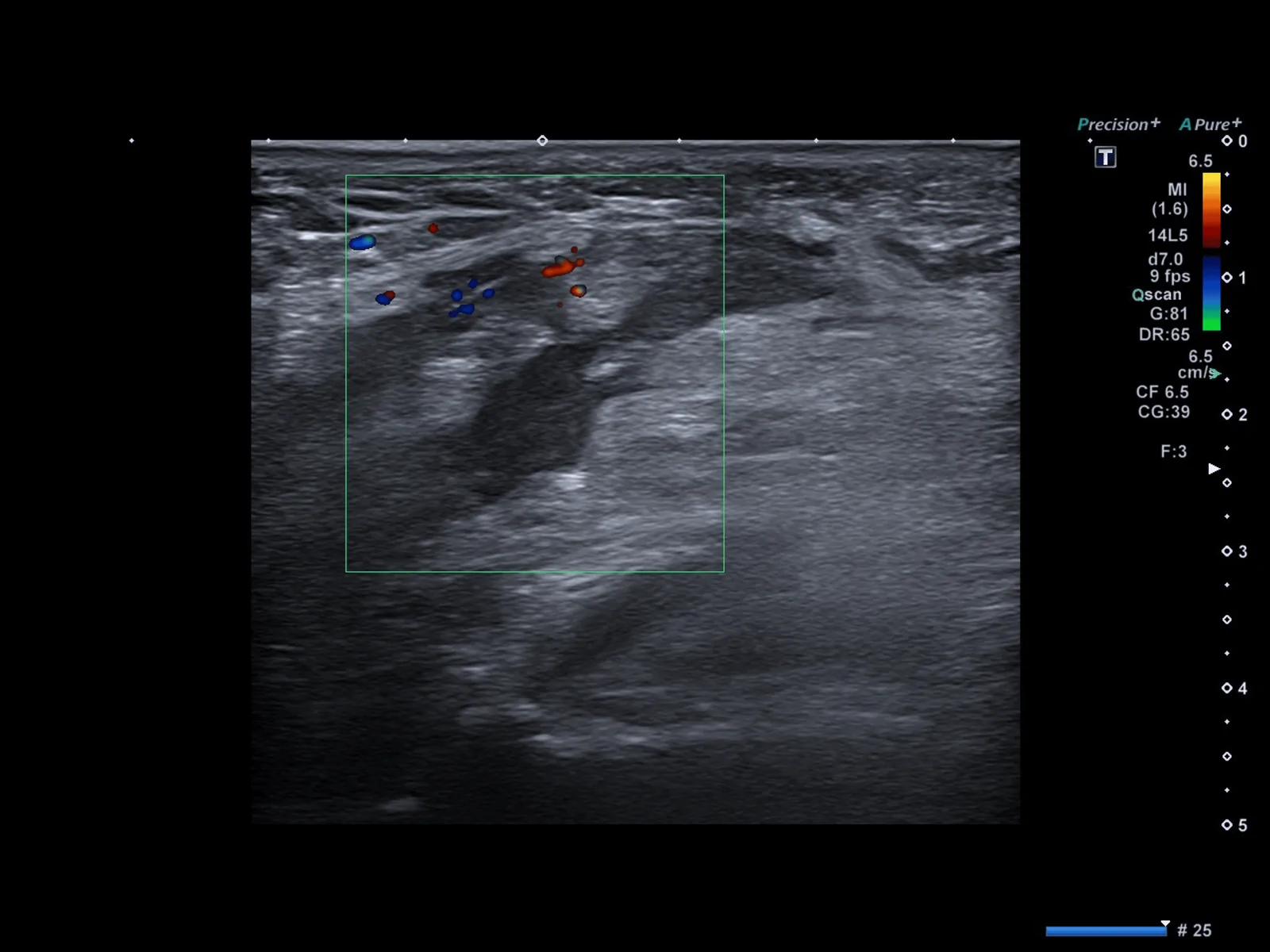
Ultrasound examination of the left inguinal region Color Doppler ultrasonography demonstrating a non-reducible left inguinoscrotal fat-containing lesion with preserved internal vascular flow and an estimated neck diameter of approximately 7 mm, initially interpreted as an incarcerated recurrent inguinal hernia. Surgical exploration subsequently demonstrated that the lesion corresponded to an abscess adjacent to the vas deferens rather than a recurrent inguinal hernia.

**Figure 2:**
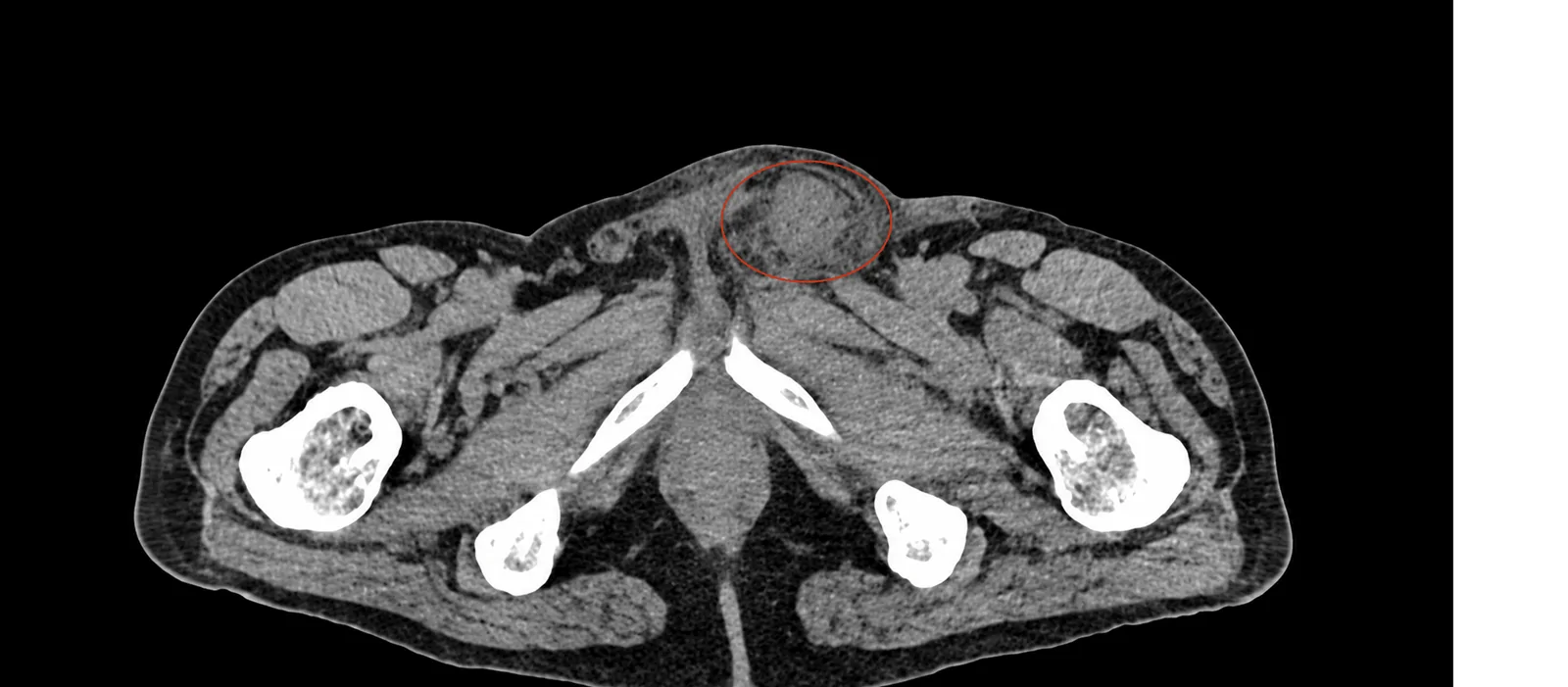
Axial non-contrast computed tomography of the left inguinal region Axial non-contrast CT image demonstrating a 27 × 28 × 53 mm fat-containing soft-tissue lesion within the left inguinal canal (red circle), initially interpreted as an incarcerated recurrent inguinal hernia. The circled lesion shows surrounding inflammatory fat stranding, contributing to the preoperative radiological diagnosis. Surgical exploration subsequently revealed that the lesion corresponded to an abscess adjacent to the vas deferens rather than a recurrent inguinal hernia.

**Figure 3:**
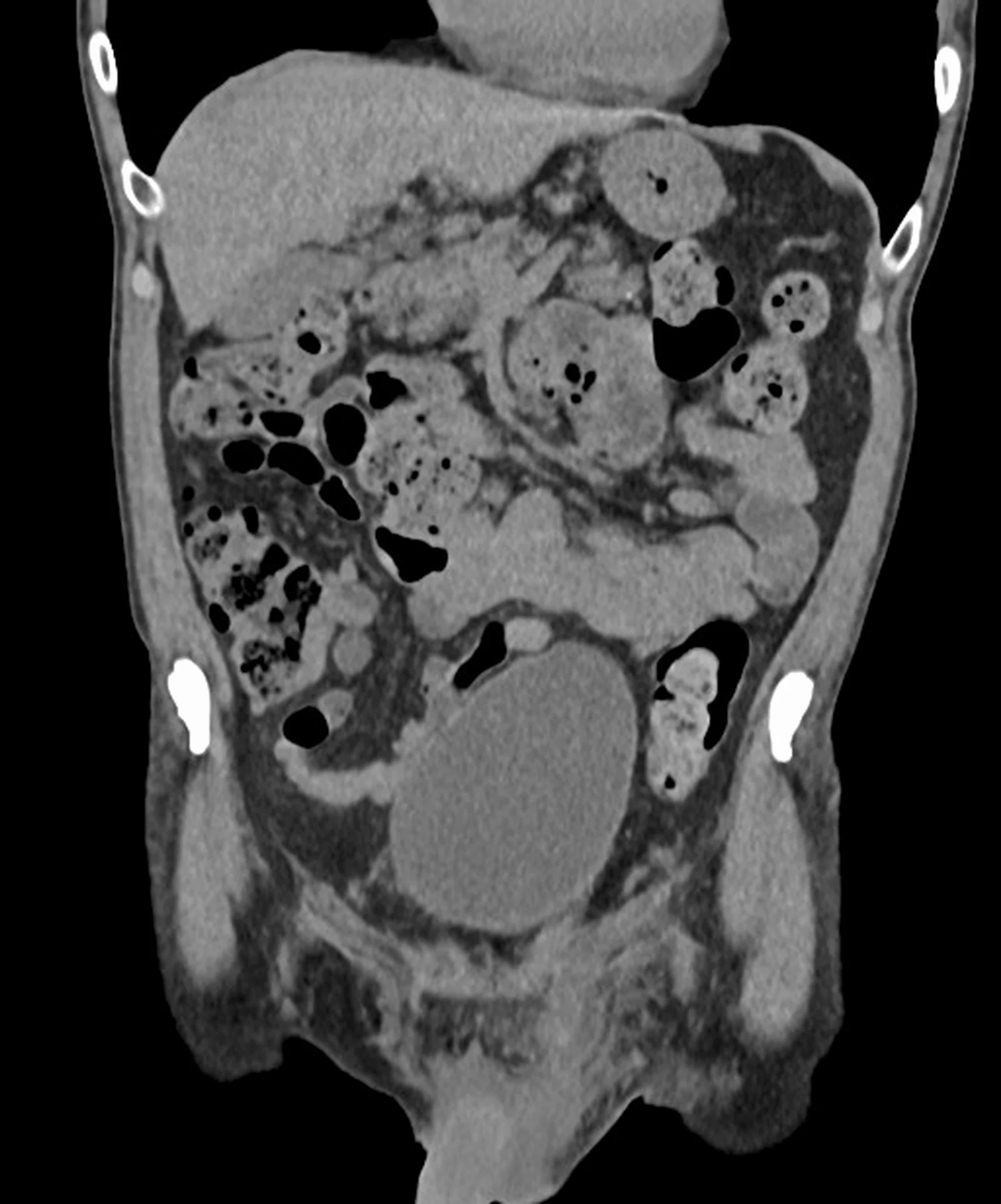
Coronal non-contrast computed tomography of the left inguinal region Coronal non-contrast computed tomography (CT) reconstruction demonstrating a 27 × 28 × 53 mm fat-containing soft-tissue lesion within the left inguinal canal, initially interpreted as an incarcerated recurrent inguinal hernia. Associated ipsilateral ureterohydronephrosis secondary to distal ureteral stenosis is also demonstrated. Surgical exploration subsequently identified the lesion as an abscess adjacent to the vas deferens rather than a recurrent inguinal hernia.

Ultrasonography, performed as the initial imaging modality, demonstrated a non-reducible left inguinoscrotal fat-containing lesion with preserved Doppler flow and an estimated neck diameter of approximately 7 mm, findings considered compatible with an incarcerated inguinal hernia (Figure 1). To further evaluate the lesion, a non-contrast computed tomography (CT) scan of the abdomen and pelvis was obtained. CT confirmed a 27 × 28 × 53 mm fat-containing left inguinal mass, which was interpreted as an incarcerated recurrent inguinal hernia (Figures 2-3). The examination also demonstrated ipsilateral ureterohydronephrosis secondary to distal ureteral stenosis without evidence of urinary calculi. As the CT examination was performed without intravenous contrast, enhancement characteristics of the lesion could not be assessed.

Because the diagnosis of an incarcerated recurrent inguinal hernia was already strongly suspected based on the clinical presentation and ultrasonographic findings, a non-contrast CT scan of the abdomen and pelvis was obtained primarily as an adjunctive preoperative examination before urgent surgery rather than as the primary diagnostic modality. Consequently, intravenous contrast was not administered.

Because of the associated ureterohydronephrosis, cystoscopy was performed and demonstrated a decompensated bladder without evidence of bladder outlet obstruction. A left double-J ureteral stent was inserted to relieve the ureteral obstruction.

Given the concordant clinical and radiological findings strongly suggestive of an incarcerated recurrent inguinal hernia requiring urgent surgical management, a minimally invasive approach was initially undertaken. Diagnostic laparoscopy was performed first to assess the viability of the intra-abdominal viscera, facilitate reduction of any incarcerated contents if present, and identify the recurrent hernia defect. However, no incarcerated bowel, omentum, recurrent hernia sac, or abdominal wall defect was identified. There was also no evidence of intra-abdominal pathology or peritoneal carcinomatosis. As the laparoscopic findings were inconsistent with the presumed diagnosis, exploration of the left inguinal region was subsequently performed. This revealed a firm necrotic inflammatory mass located adjacent to the vas deferens, without involvement of the testis or spermatic cord. The lesion was completely excised. As no recurrent hernia or fascial defect was identified, neither hernia repair nor mesh implantation was performed.

**Figure 4:**
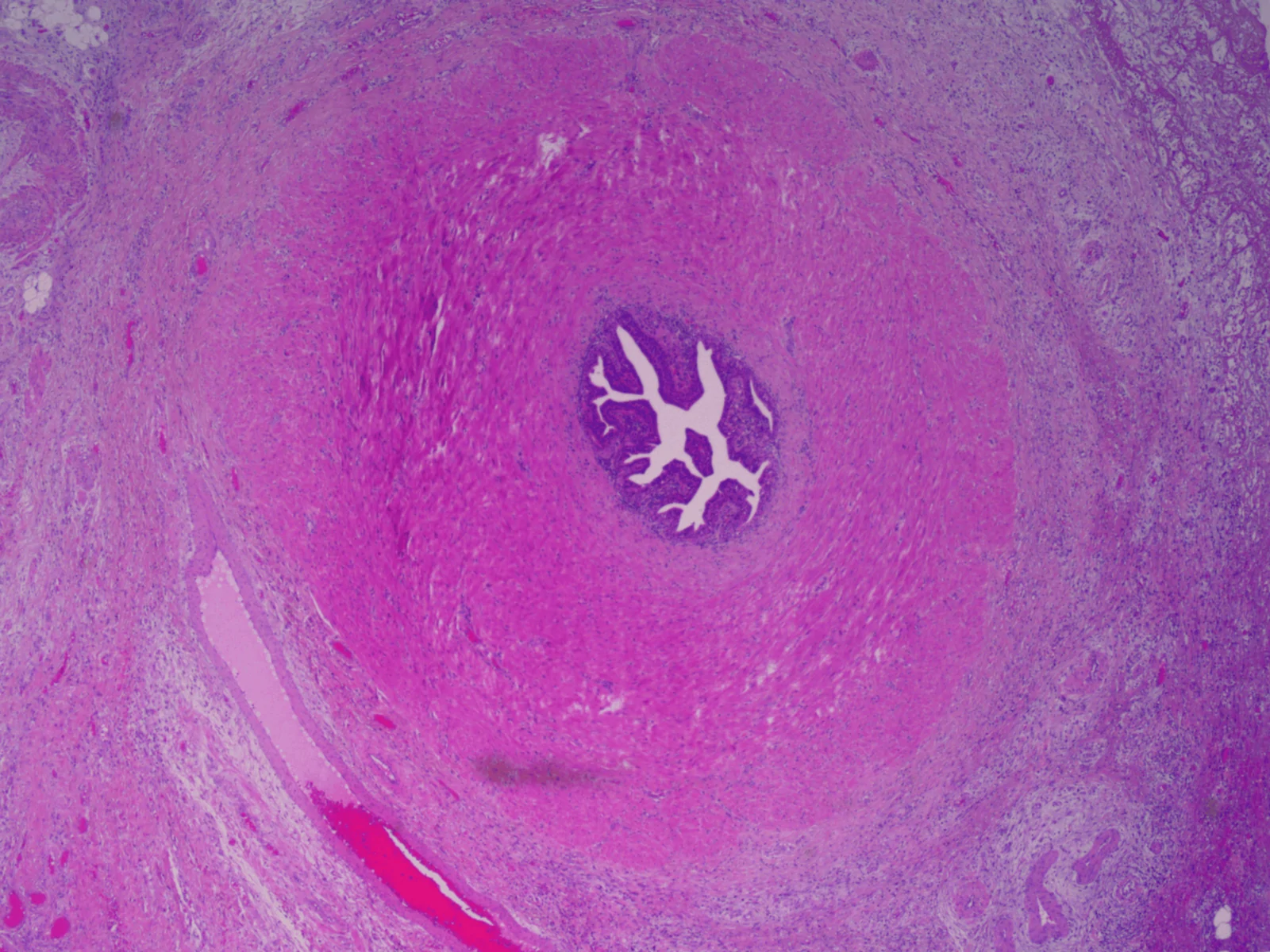
Histopathological examination of the vas deferens and adjacent inflammatory tissue Low-power photomicrograph (hematoxylin and eosin stain, ×5 magnification) demonstrating acute suppurative inflammation and abscess formation in the fibrofatty tissue adjacent to the vas deferens. The wall of the vas deferens shows no histopathological evidence of inflammatory involvement. No evidence of malignancy or granulomatous inflammation is identified.

**Figure 5:**
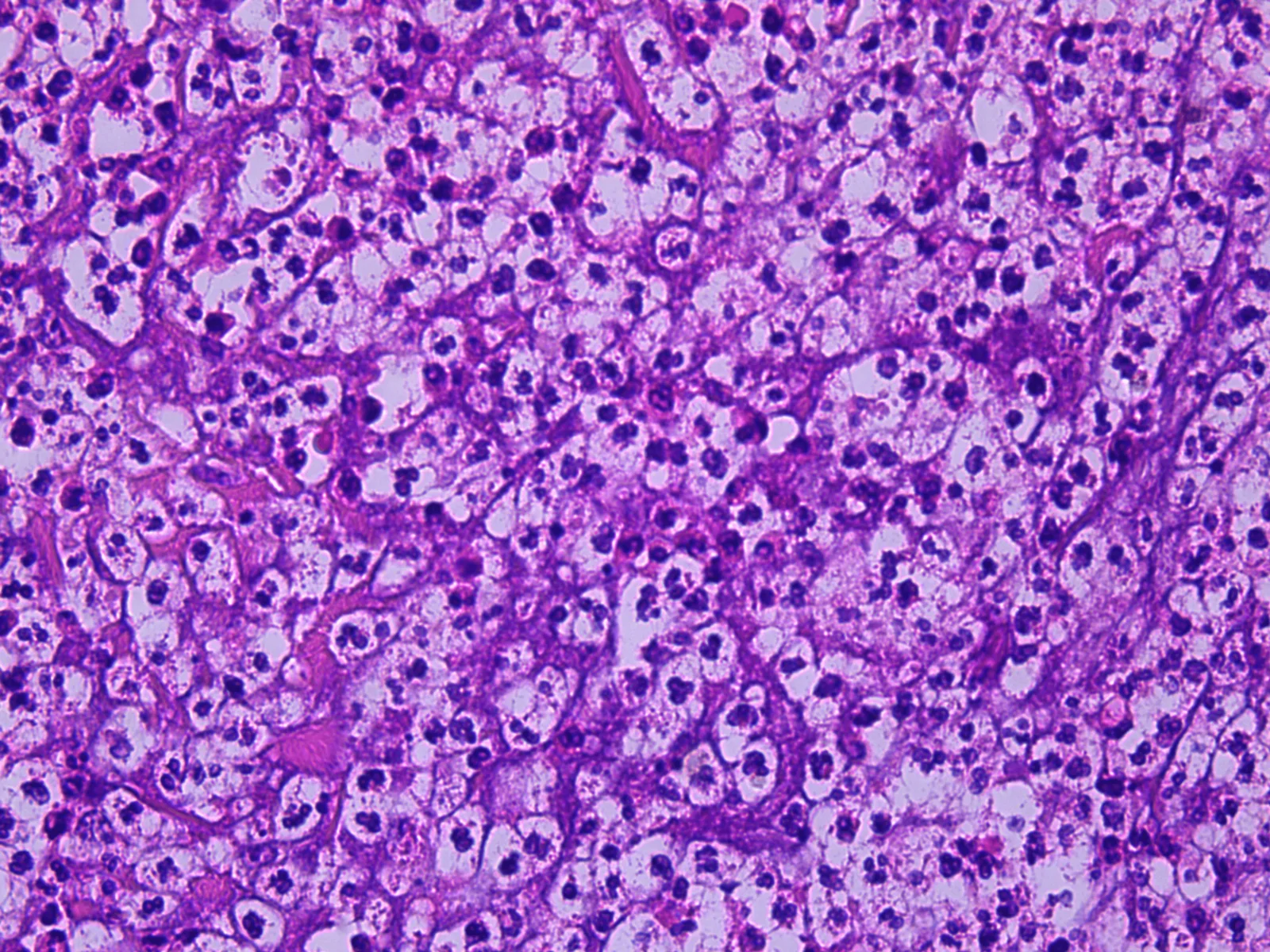
High-power histopathological view of the abscess adjacent to the vas deferens High-power photomicrograph (hematoxylin and eosin stain, ×40 magnification) demonstrating dense acute suppurative inflammatory infiltrate composed predominantly of neutrophils with abundant necrotic cellular debris, consistent with abscess formation. No evidence of malignancy is identified.

Histopathological examination demonstrated a 4.5 × 3 × 2.9 cm specimen composed of fibrofatty tissue with extensive necrosis, including a 3 cm segment of the vas deferens (Figure 4). Microscopic analysis confirmed a deep acute abscess adjacent to the vas deferens, without histological involvement of the vas deferens wall and without evidence of malignancy or granulomatous disease (Figure 5).

Microbiological analysis of the intraoperative specimen yielded Enterococcus faecalis, susceptible to amoxicillin, vancomycin, and high-level gentamicin synergy, while anaerobic cultures remained sterile. The patient received seven days of intravenous amoxicillin-clavulanic acid followed by a seven-day course of oral amoxicillin-clavulanic acid.

The postoperative course was uneventful, with progressive normalization of inflammatory markers and complete resolution of symptoms. At the 18-month follow-up, the patient remained asymptomatic, with no clinical evidence of recurrence.

No urinary calculi were identified on imaging. A double-J ureteral stent was inserted during cystoscopy and was removed one month later during outpatient urological follow-up. The patient's urinary symptoms resolved completely. A follow-up CT scan performed two months after surgery demonstrated complete resolution of the left ureterohydronephrosis. Although a definitive causal relationship cannot be established, these findings support the hypothesis that the distal ureteral stenosis and associated ureterohydronephrosis were secondary to local inflammatory changes and extrinsic compression caused by the adjacent inflammatory process rather than primary ureteral disease.

## Discussion

A focused literature search was conducted using PubMed/MEDLINE to identify reports of acute inflammatory or suppurative conditions involving the vas deferens, adjacent peri-deferential tissues, or spermatic cord presenting as inguinal or groin masses.

The search included articles published up to the time of manuscript preparation, without time restriction. The following search strategy was used: (vasitis OR "vas deferens" OR deferentitis OR "spermatic cord" OR funiculitis) AND (abscess OR infection OR suppurat OR phlegmon) AND (inguinal OR groin OR hernia OR "inguinal mass" OR incarcerated OR strangulated OR mimick OR masquerad*).

The initial search yielded 100 articles. After applying the filters "English" and "Humans", 54 articles remained. Titles and abstracts were screened for relevance, and nine articles were selected for full-text review. Reference lists of the selected articles were manually screened, leading to the identification of two additional relevant studies. A total of 11 articles were ultimately included in the final analysis (Table 1).

**Table 1:** Summary of reported cases and reviews of vas deferens and spermatic cord pathologies mimicking inguinal hernia This table summarizes published case reports and review articles describing inflammatory, infectious, and neoplastic conditions involving the vas deferens or spermatic cord that presented as inguinal or groin masses mimicking inguinal hernia. Data include patient demographics, diagnosis, and key clinical and imaging findings. CT - computed tomography; US - ultrasonography; MRI - magnetic resonance imaging; PMID - PubMed identification number; NR - not reported; M - male

Author (year)	PMID	Article type	Age	Sex	Pathology / diagnosis	Key findings
Al-Gburi et al., 2023 [5]	38288181	Case report	40	M	Acute vasitis	CT demonstrated inflammatory thickening of the vas deferens, avoiding unnecessary surgery for suspected incarcerated hernia.
Ryan & Harte, 1988 [4]	3347975	Case report	25	M	Suppurative vasitis	Painful inguinal mass initially diagnosed as hernia; surgery revealed a vas deferens abscess.
Yam & Ng, 2014 [6]	25096656	Case report	54	M	Spermatic cord abscess	Diabetic patient with epididymo-orchitis; CT and US identified a spermatic cord abscess requiring drainage and antibiotics.
Akintayo et al., 2022 [8]	36204416	Case report	49	M	Spermatic cord abscess	Diabetic patient with spermatic cord and perinephric abscesses managed by incision, drainage, and antibiotics.
Augustin & Kunjko, 2015 [9]	25494476	Case report	70	M	Spermatic cord abscess	Post-coronary angiography abscess mimicked incarcerated hernia; treated with excision and antibiotics.
Machida et al., 2008 [7]	18301983	Case report	81	M	Spermatic cord abscess	CT suggested strangulated hernia; surgery revealed a spermatic cord abscess associated with a prostatic abscess.
Bhosale et al., 2008 [1]	18480486	Review	NR	NR	Imaging review	Reviews benign, inflammatory, infectious, and malignant inguinal masses and their imaging features.
Park et al., 2016 [2]	27209238	Review	NR	NR	Sonographic review	Ultrasound is the first-line modality for differentiating inguinal hernias from their mimics.
Revzin et al., 2016 [3]	27715712	Review	NR	NR	Imaging review	Comprehensive review of ultrasound, CT, and MRI findings in inguinal canal pathology.
Horn et al., 2001 [10]	11730227	Case-based article	71, 54	M	Dilated spermatic cord veins	Two cirrhotic patients with hernia mimics diagnosed using Doppler ultrasound.
Ahmed et al., 2022 [11]	35337334	Case report	65	M	Leiomyosarcoma of the spermatic cord	Malignant spermatic cord tumor presenting as an inguinoscrotal mass requiring orchiectomy.

Although incarcerated or strangulated inguinal hernia remains the most common cause of acute groin swelling, numerous inflammatory, infectious, vascular, and neoplastic conditions may present with an identical clinical picture, making the differential diagnosis particularly challenging [1-3].

Analysis of the selected studies demonstrates a consistent overlap in clinical presentation, with most patients presenting with groin pain, swelling, and a palpable mass, frequently leading to an initial misdiagnosis of incarcerated inguinal hernia. Among infectious causes, vasitis, funiculitis, and spermatic cord or peri-deferential abscesses represent rare but clinically significant entities. Early reports described suppurative inflammation of the vas deferens presenting as an inguinal mass [4], and more recent studies confirm that these conditions remain underrecognized [5].

Progression from inflammation to abscess formation represents a more advanced stage within the same pathological spectrum. Several reports describe spermatic cord abscess associated with epididymo-orchitis, prostatic abscess, or systemic infection [6-8]. In some cases, infection may be iatrogenic, particularly following vascular or urological procedures [9]. Identified risk factors include diabetes mellitus, chronic kidney disease, genitourinary infection, and previous invasive interventions [6-9], suggesting that both host-related factors and ascending infectious spread contribute to disease development.

The present patient accumulated several potential predisposing factors for ascending genitourinary infection, including recurrent prostatitis, previous prostate surgery, and ipsilateral distal ureteral obstruction. Intraoperative cultures yielded *Enterococcus faecalis*, a microorganism commonly associated with urinary tract and prostatic infections, supporting a probable genitourinary origin of the infectious process. Although a definite causal relationship cannot be established, these findings suggest that local extension from the lower urinary tract may have contributed to the development of the abscess adjacent to the vas deferens.

In addition to infectious causes, other rare conditions may mimic inguinal hernia. Vascular abnormalities have been reported, particularly in cirrhotic patients [10]. Furthermore, neoplastic lesions must be considered. Ahmed et al. described a rare case of leiomyosarcoma of the spermatic cord presenting as an inguinoscrotal mass, highlighting that malignant tumors, although uncommon, may clinically mimic hernia and should be included in the differential diagnosis [11].

From a diagnostic perspective, ultrasonography remains the first-line imaging modality for evaluating inguinal swelling, whereas computed tomography is valuable when the diagnosis is uncertain or when deeper extension is suspected [1-3,5-8]. In the present case, both ultrasonography and non-contrast CT suggested an incarcerated recurrent inguinal hernia. However, the abscesses exhibited predominantly fat attenuation with surrounding inflammatory fat stranding and were therefore interpreted as herniated omental fat. Because intravenous contrast was not administered, enhancement characteristics that might have suggested an abscess could not be assessed. This case illustrates an important diagnostic pitfall and highlights that inflammatory lesions adjacent to the vas deferens may closely mimic an incarcerated inguinal hernia on imaging.

The associated distal ureteral stenosis and ipsilateral ureterohydronephrosis represented another unusual finding. In the absence of urinary calculi, these abnormalities were considered most likely secondary to local inflammatory changes and extrinsic compression caused by the adjacent infectious process, although a pre-existing ureteral abnormality cannot be completely excluded.

Management depends on disease severity. While uncomplicated inflammatory conditions such as vasitis may be successfully managed with antibiotic therapy alone [5], abscesses generally require drainage or complete surgical excision combined with culture-guided antimicrobial therapy [6-9]. Histopathological examination remains essential, particularly when malignancy cannot be excluded [11].

To our knowledge, this represents one of the very few reported cases of an abscess adjacent to the vas deferens presenting as an incarcerated recurrent inguinal hernia. This case emphasizes three important learning points. First, inflammatory peri-deferential lesions may closely mimic fat-containing inguinal hernia on ultrasonography and CT. Second, previous urological disease, including recurrent prostatitis or prior prostate surgery, should raise suspicion for an underlying infectious process. Finally, definitive diagnosis frequently relies on surgical exploration with histopathological and microbiological confirmation.

## Conclusions

Abscess adjacent to the vas deferens is an exceptionally rare cause of acute groin swelling that may closely mimic an incarcerated recurrent inguinal hernia. This case highlights three important learning points: inflammatory peri-deferential phlegmon may be misinterpreted as a fat-containing hernia on imaging; previous urological disease should raise suspicion for an underlying infectious process; and atypical imaging or intraoperative findings should prompt consideration of alternative non-hernia diagnoses. Early recognition may facilitate appropriate surgical management and culture-guided antimicrobial therapy.
